# Trends and hotspots in research on medical images with deep learning: a bibliometric analysis from 2013 to 2023

**DOI:** 10.3389/frai.2023.1289669

**Published:** 2023-11-09

**Authors:** Borui Chen, Jing Jin, Haichao Liu, Zhengyu Yang, Haoming Zhu, Yu Wang, Jianping Lin, Shizhong Wang, Shaoqing Chen

**Affiliations:** ^1^First School of Clinical Medicine, Fujian University of Traditional Chinese Medicine, Fuzhou, China; ^2^College of Rehabilitation Medicine, Fujian University of Traditional Chinese Medicine, Fuzhou, China; ^3^The School of Health, Fujian Medical University, Fuzhou, China

**Keywords:** deep learning, medical images, bibliometric analysis, CiteSpace, trends, hotspots

## Abstract

**Background:**

With the rapid development of the internet, the improvement of computer capabilities, and the continuous advancement of algorithms, deep learning has developed rapidly in recent years and has been widely applied in many fields. Previous studies have shown that deep learning has an excellent performance in image processing, and deep learning-based medical image processing may help solve the difficulties faced by traditional medical image processing. This technology has attracted the attention of many scholars in the fields of computer science and medicine. This study mainly summarizes the knowledge structure of deep learning-based medical image processing research through bibliometric analysis and explores the research hotspots and possible development trends in this field.

**Methods:**

Retrieve the Web of Science Core Collection database using the search terms “deep learning,” “medical image processing,” and their synonyms. Use CiteSpace for visual analysis of authors, institutions, countries, keywords, co-cited references, co-cited authors, and co-cited journals.

**Results:**

The analysis was conducted on 562 highly cited papers retrieved from the database. The trend chart of the annual publication volume shows an upward trend. Pheng-Ann Heng, Hao Chen, and Klaus Hermann Maier-Hein are among the active authors in this field. Chinese Academy of Sciences has the highest number of publications, while the institution with the highest centrality is Stanford University. The United States has the highest number of publications, followed by China. The most frequent keyword is “Deep Learning,” and the highest centrality keyword is “Algorithm.” The most cited author is Kaiming He, and the author with the highest centrality is Yoshua Bengio.

**Conclusion:**

The application of deep learning in medical image processing is becoming increasingly common, and there are many active authors, institutions, and countries in this field. Current research in medical image processing mainly focuses on deep learning, convolutional neural networks, classification, diagnosis, segmentation, image, algorithm, and artificial intelligence. The research focus and trends are gradually shifting toward more complex and systematic directions, and deep learning technology will continue to play an important role.

## 1. Introduction

The origin of radiology can be seen as the beginning of medical image processing. The discovery of X-rays by Röntgen and its successful application in clinical practice ended the era of disease diagnosis relying solely on the clinical experience of doctors (Glasser, 1995). The production of medical images provides doctors with more data, enabling them to diagnose and treat diseases more accurately. With the continuous improvement of computer performance and image processing technology represented by central processing units (CPUs; Dessy, 1976), medical image processing has become more efficient and accurate in medical research and clinical applications. Initially, medical image processing was mainly used in medical imaging diagnosis, such as analyzing and diagnosing X-rays, CT, MRI, and other images. Nowadays, medical image processing has become an important research tool in fields such as radiology, pathology, and biomedical engineering, providing strong support for medical research and clinical diagnosis (Hosny et al., 2018; Hu et al., 2022; Lin et al., 2022).

Deep learning originated from artificial neural networks, which can be traced back to the 1940 and 1950s when scientists proposed the perceptron model and neuron model to simulate the working principles of human nervous system (Rosenblatt, 1958; McCulloch and Pitts, 1990). However, limited by the weak performance of computers at that time, these models were quickly abandoned. In 2006, Canadian computer scientist Geoffrey Hinton and his team proposed a model called “deep belief network,” which adopted a deep structure and solved the shortcomings of traditional neural networks. This is considered as the starting point of deep learning (Hinton et al., 2006).

In recent years, with the rapid development of the Internet, massive data are constantly generated and accumulated, which are very favorable for deep learning networks that require a large amount of data for training (Misra et al., 2022). Additionally, the development of computer devices such as graphics processing units (GPUs) and tensor processing units(TPUs) has made the training of deep learning models faster and more efficient (Alzubaidi et al., 2021; Elnaggar et al., 2022). Furthermore, the continuous improvement and optimization of deep learning algorithms have also led to the continuous improvement of the performance of deep learning models (Minaee et al., 2022). Therefore, the application of deep learning is becoming more and more widespread in various fields, including medical image processing.

Deep learning has many advantages in processing medical images. Firstly, it does not require human intervention and can automatically learn and extract features, achieving automation in processing (Yin et al., 2021). Secondly, it can process a large amount of data simultaneously, with processing efficiency far exceeding traditional manual methods (Narin et al., 2021). Thirdly, its accuracy is also high, able to learn more complex features and discover subtle changes and patterns that are difficult for humans to perceive (Han et al., 2022). Lastly, it is less affected by subjective human factors, leading to relatively more objective results (Kerr et al., 2022).

Bibliometrics is a quantitative method for evaluating the research achievements of researchers, institutions, countries, or subject areas, and can be traced back to the 1960s (Schoenbach and Garfield, 1956). In bibliometric analysis, the citation half-life of an article has two characteristics: first, classical articles are continuously cited; second, some articles are frequently cited within a certain period and quickly reach a peak. The length of time that classical articles are continuously cited is closely related to the speed of development of basic research, while the frequent citation of certain articles within a specific period represents the dynamic changes in the corresponding field. Generally speaking, articles that reflect dynamic changes in the field are more common than classical articles. In Web of Science, papers that are cited in one or more fields and rank in the top 1% of citation counts for their publication year are included as highly cited papers. Visual analysis of highly cited papers is more effective in identifying popular research areas and trends compared to visual analysis of all search results. CiteSpace is a visualization software that employs bibliometric methods, developed by Professor Chaomei Chen at Drexel University (Chen, 2006).

Therefore, to gain a deeper understanding of the research hotspots and possible development trends of deep learning-based medical image processing, this study aims to analyze highly cited papers published between 2013 and 2023 using bibliometric methods, intends to identify the authors, institutions, and countries with the most research achievements, and provide an overall review of the knowledge structure among the highly cited papers. Expected to be helpful for researchers in this field.

## 2. Methods

### 2.1. Search strategy and data source

A search was conducted in the Web of Science Core Collection database using the search terms “deep learning” and “medical imaging,” along with their synonyms and related terms. The complete search string is as follows: (TS = Deep Learning OR “Deep Neural Networks” OR “Deep Machine Learning” OR “Deep Artificial Neural Networks” OR “Deep Models” OR “Hierarchical Learning” OR “Deep architectures” OR “Multi-layer Neural Networks” OR “Large-scale Neural Networks” OR “Deep Belief Networks”) AND (TS = “Medical imaging” OR “Radiology imaging” OR “Diagnostic imaging” OR “Clinical imaging” OR “Biomedical imaging” OR “Radiographic imaging” OR “Tomographic imaging” OR “Imaging modalities” OR “Medical visualization” OR “Medical image analysis”). The search was refined to include only articles published between 2013 and 2023, with a focus on highly cited papers. The search yielded a total of 562 results. The article type was restricted to papers, and the language was limited to English.

### 2.2. Scientometric analysis methods

Due to the Web of Science export limitation, the record options were set to export records 1–500 and 501–562 separately, and the record content including full records and cited references. This plain text file served as the source file for the analysis. Next, a new project was established in CiteSpace 6.1.R6, with the project location and data storage location set up. The input and output function of CiteSpace were used to convert the plain text file into a format that could be analyzed in CiteSpace. The remaining parameters were set as follows: the time slicing was set from 2013 to 2023, with a yearly time interval; the node types selected included authors, institutions, countries keyword, co-cited references, co-cited authors, and co-cited journals; the threshold for “Top N,” “Top N%,” and “g-index” were set to default; the network pruning was set to pathfinder and pruning the merged network; the visualization was set to static cluster view and show merged network to display the overall network.

In the map generated by CiteSpace, there are multiple elements. The various nodes available for analysis are represented as circles on the map, with their size generally indicating the quantity—the larger the circle, the greater the quantity. The circles are composed of annual rings, with the color of each ring representing the year, and the thickness of the ring determined by the number of corresponding nodes in that year. The more nodes in a year, the thicker the ring. The meaning of the “Centrality” option in CiteSpace menu is “Betweenness Centrality” (Chen, 2005). CiteSpace utilizes this metric to discover and measure the importance of nodes, and highlights nodes with purple circles when the centrality greater than or equal to 0.1. It means that only nodes with centrality greater than or equal to 0.1 are worth emphasizing their importance. The calculation method is based on the formulation introduced by Freeman (1977), and the formula is as follows:


$$BC_{i}=\underset{s\neq i\neq t}{\sum }\frac{n_{st}^{i}}{g_{st}}$$

In this formula, $g_{st}$ represents the number of shortest paths from node s to node t , and $n_{st}^{i}$ represents the number of those shortest paths from node s to node t that pass through node i . From the information transmission perspective, the higher the Betweenness Centrality, the greater the importance of the node. Removing these nodes will have a larger impact on network transmission.

## 3. Results

### 3.1. Analysis of annual publication volume

**Figure 1:**
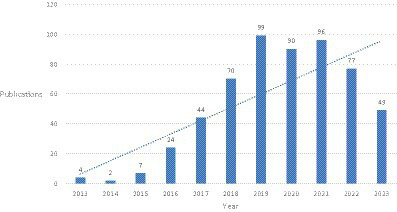
Annual quantitative distribution of publications.

The trend of annual publication volume shows that from 2013 to 2023, the number of related studies fluctuated slightly each year but showed an overall upward trend. Overall, it can be divided into three stages: before 2016, the number of papers was relatively small; after 2016, the number of papers increased year by year, and the rate of increase accelerated. From 2016 to 2019, there was an increase of about 20 papers per year on the basis of the previous year. After 2019, the growth rate slowed down, but there was still a high level of publications each year (Figure 1).

### 3.2. Analysis of authors

**Figure 2:**
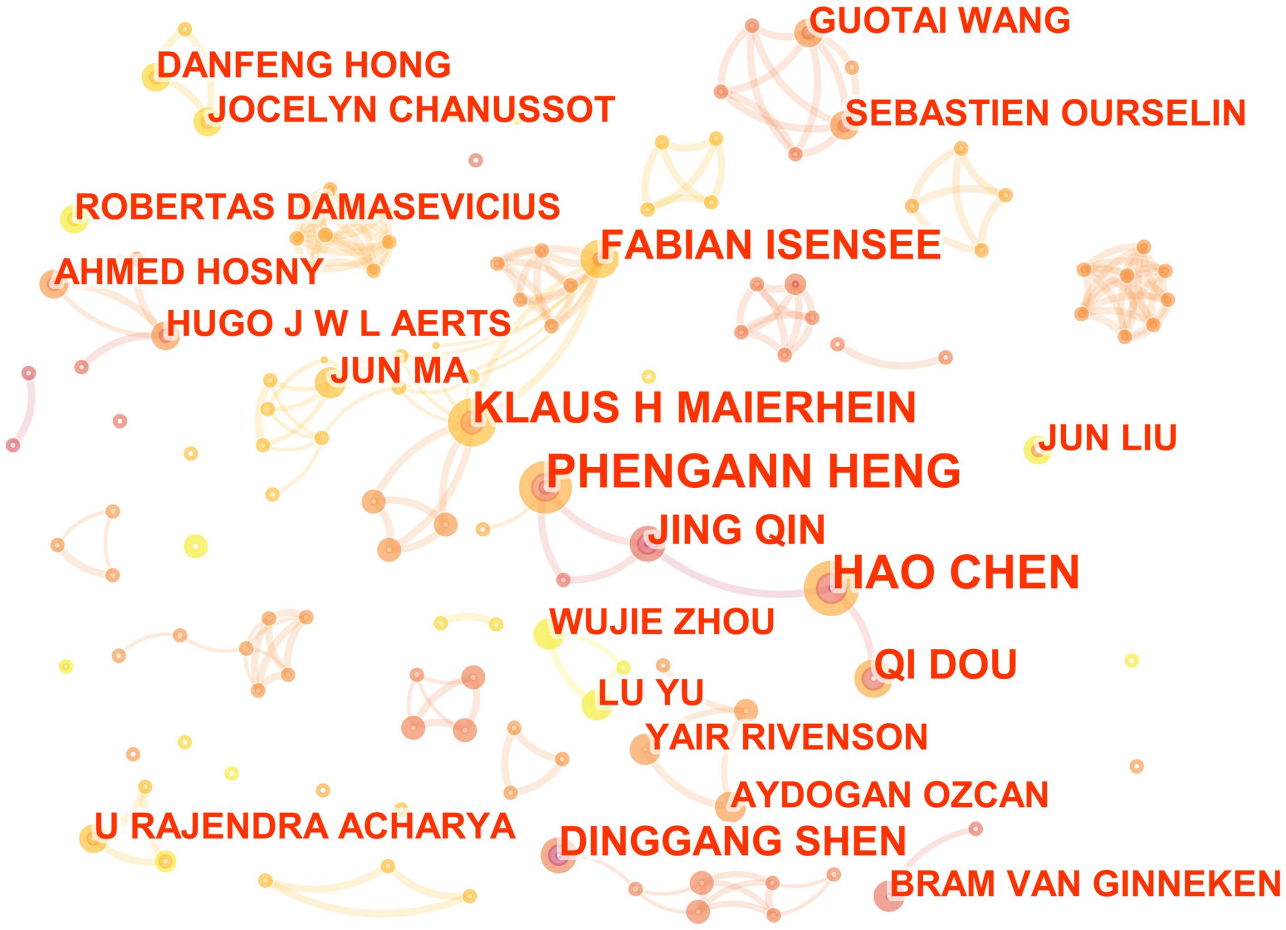
The collaborative relationship map of researchers in the field of medical image processing with deep learning from 2013 to 2023.The size of nodes represents the number of papers published by the author. The links between nodes reflect the strength of collaboration.

Among the 562 articles included, there are a total of 364 authors (Figure 2). Pheng-Ann Heng and Hao Chen ranks first with seven publications, Klaus Hermann Maier-Hein ranks second with six publications, while Fabian Isensee, Jing Qin, Qi Dou, and Dinggang Shen are tied for third place with five publications each. From Figure 2, it can be seen that there are many small groups of authors, but no very large research groups, and there are still many authors who do not have any collaborative relationships with each other.

### 3.3. Analysis of institutions

**Figure 3:**
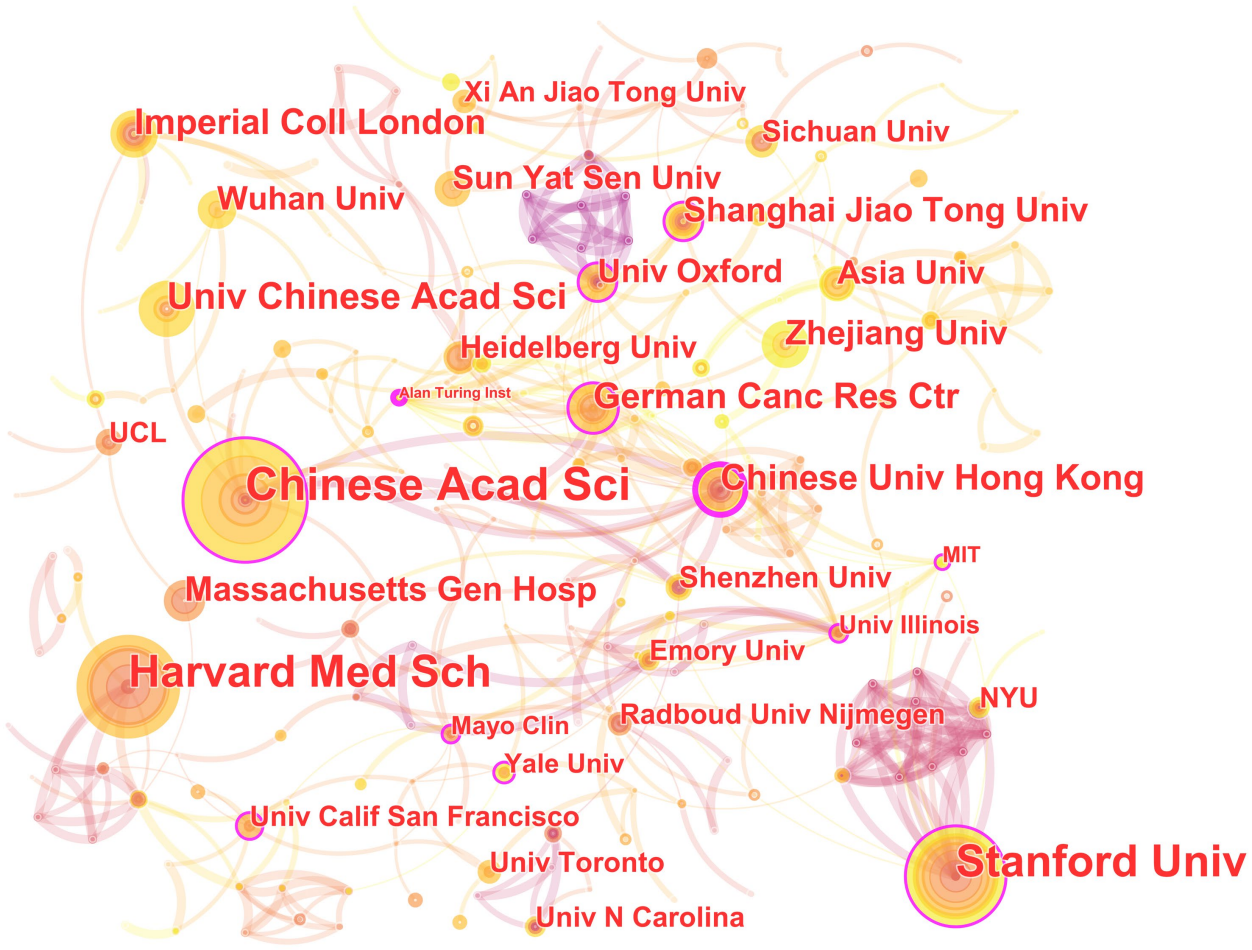
The collaborative relationship map of institutions in the field of medical image processing with deep learning from 2013 to 2023. The size of nodes represents the number of papers published by the institution. The links between nodes reflect the strength of collaboration.

In the 562 papers included, there are a total of 311 institutions (Figure 3; Table 1). The institution with the highest publication output is Chinese Academy of Sciences, and the institution with the highest centrality is Stanford University. The map shows that there are close collaborative relationships between institutions, but these relationships are based on one or more institutions with high publication output and centrality. There is less collaboration between institutions with low publication output and no centrality. As shown in Table 1, there is no necessary relationship between publication output and centrality, and the institution with the highest publication output does not necessarily have the highest centrality.

**Table 1:** Top 10 institutions by publication volume and centrality.

Rank	Number of publications	Institution	Centrality	Institution
1	32	Chinese Academy of Sciences	0.23	Stanford University
2	26	Stanford University	0.16	Chinese Academy of Sciences
3	26	Harvard Medical School	0.15	Harvard Medical School
4	14	University of Chinese Academy of Sciences	0.1	The Chinese University of Hong Kong
5	13	German Cancer Research Center	0.1	University of Oxford
6	13	The Chinese University of Hong Kong	0.08	The University of Sydney
7	13	Imperial College London	0.07	German Cancer Research Center
8	12	Zhejiang University	0.06	Shanghai Jiao Tong University
9	11	Shanghai Jiao Tong University	0.06	Asia University
10	11	Massachusetts General Hospital	0.06	University of Illinois

### 3.4. Analysis of countries

**Figure 4:**
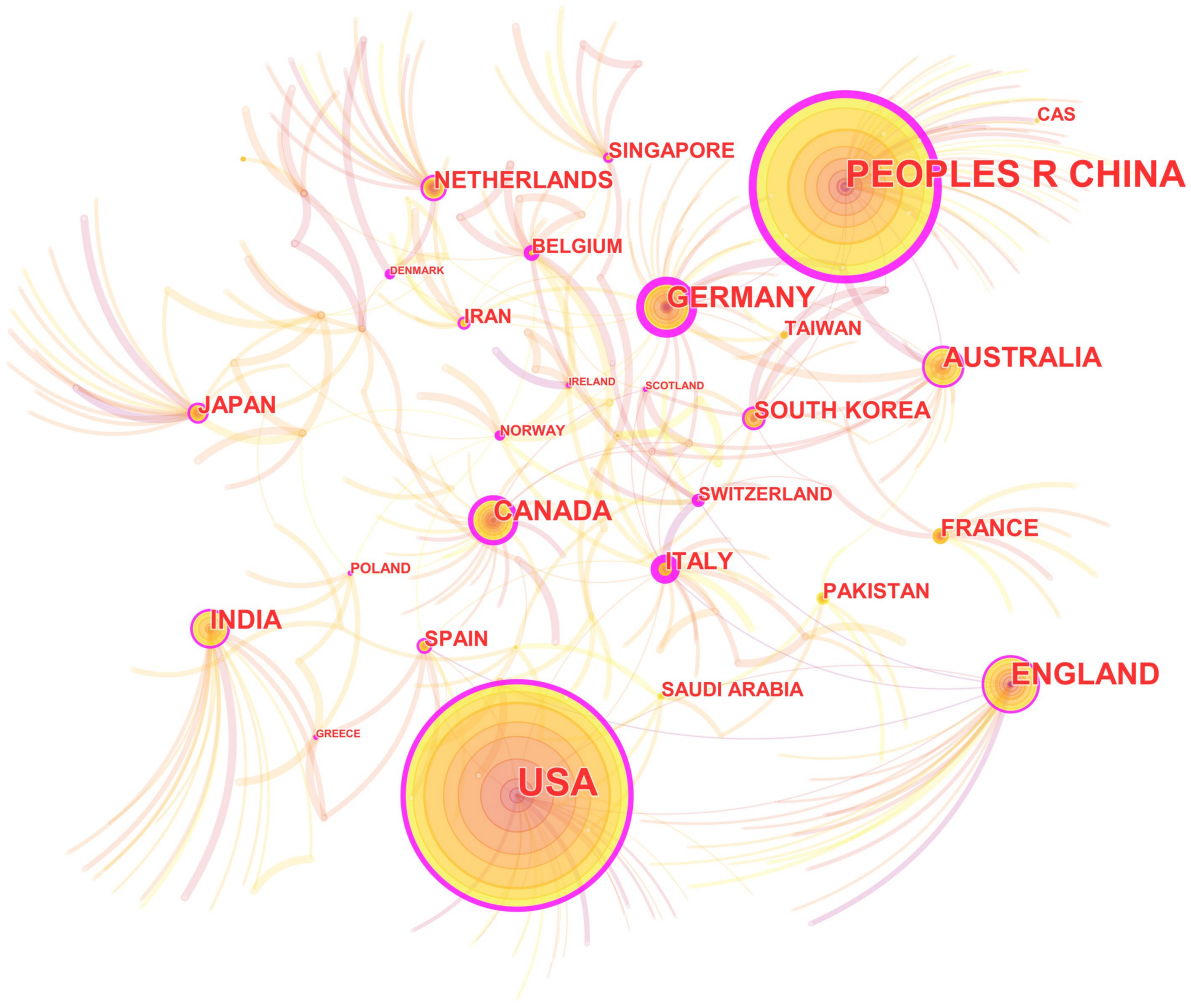
The collaborative relationship map of countries in the field of medical image processing with deep learning from 2013 to 2023. The size of nodes represents the number of papers published by the country. The links between nodes reflect the strength of collaboration.

In the 562 included papers, there are a total of 62 countries represented (Figure 4; Table 2). The United States has the highest publication output, while Germany has the highest centrality. The map shows that all countries have at least some collaboration with other countries. In general, there are three situations: some countries have a high publication output and centrality; some have a low publication output but high centrality, and some have a high publication output but low centrality.

**Table 2:** Top 10 countries by publication volume and centrality.

Rank	Number of publications	Country	Centrality	Country
1	222	United States	0.56	Germany
2	173	China	0.52	China
3	61	England	0.43	Italy
4	50	Germany	0.3	Belgium
5	44	Canada	0.25	Norway
6	43	Australia	0.22	United States
7	39	India	0.22	Switzerland
8	27	Netherlands	0.22	Denmark
9	25	France	0.21	Canada
10	24	South Korea	0.2	England

### 3.5. Analysis of keywords

**Figure 5:**
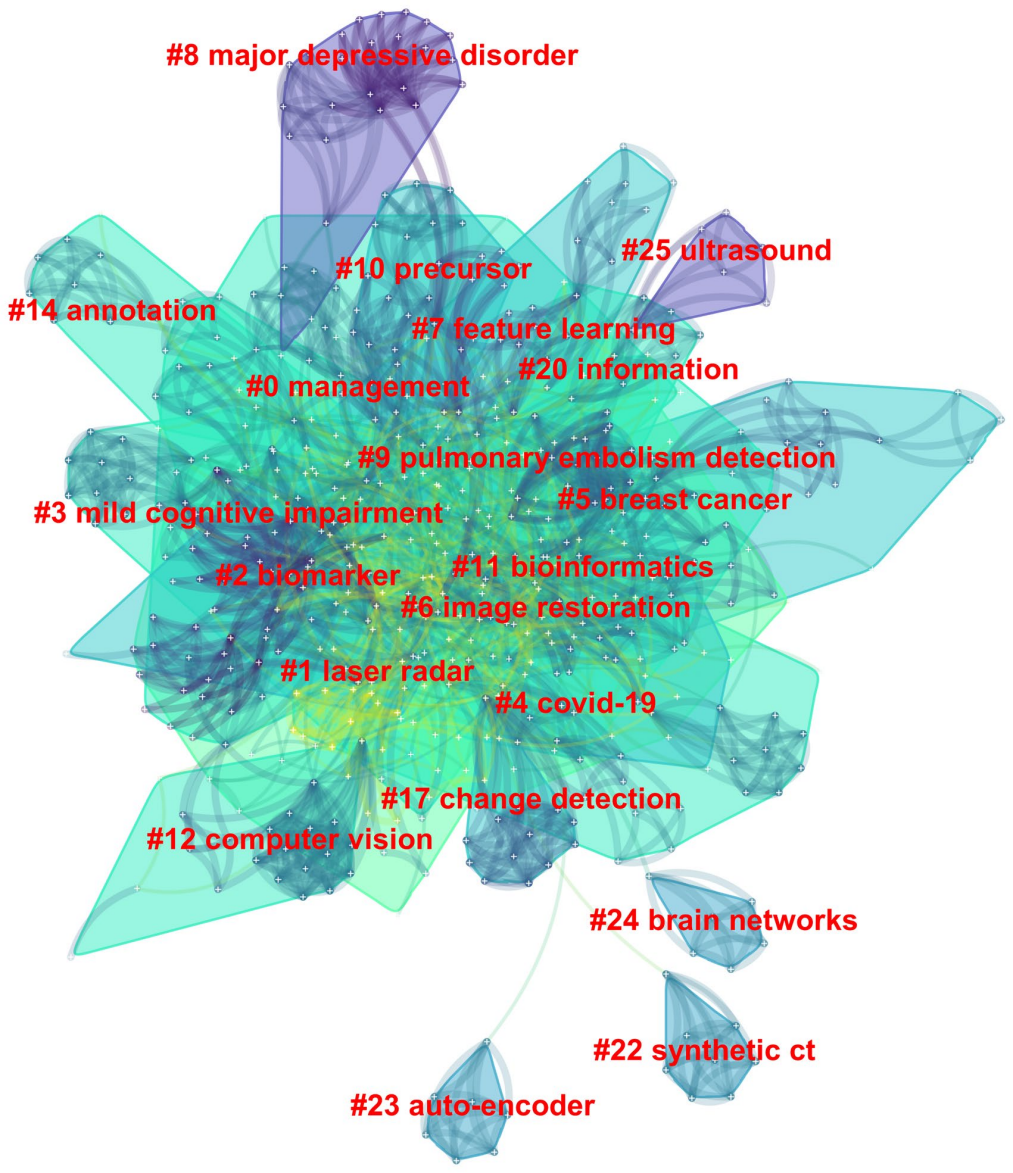
The clustering map of keywords in the field of medical image processing with deep learning from 2013 to 2023. The smaller the cluster number, the larger its size, and the more keywords it contains.

Among the 562 papers included, there were a total of 425 keywords (Figure 5; Table 3). The most frequently occurring keyword is “Deep Learning,” and the one with the highest centrality is “algorithm.” Clustering analysis of the keywords resulted in 20 clusters: management, laser radar, biomarker, mild cognitive impairment, COVID-19, image restoration, breast cancer, feature learning, major depressive disorder, pulmonary embolism detection, precursor, bioinformatics, computer vision, annotation, change detection, information, synthetic CT, auto-encoder, brain networks, and ultrasound.

**Table 3:** Top 10 keywords by quantity and centrality.

Rank	Frequency of appearances	Keyword	Centrality	Keyword
1	229	Deep learning	0.14	Algorithm
2	123	Classification	0.14	Classification
3	108	Convolutional neural network	0.12	Cancer
4	66	Segmentation	0.12	MRI
5	56	COVID-19	0.1	Convolutional neural network
6	54	Diagnosis	0.1	Machine learning
7	47	Neural network	0.08	Deep learning
8	47	Machine learning	0.08	CT
9	41	Image	0.07	Segmentation
10	39	Artificial intelligence	0.07	Model

**Figure 6:**
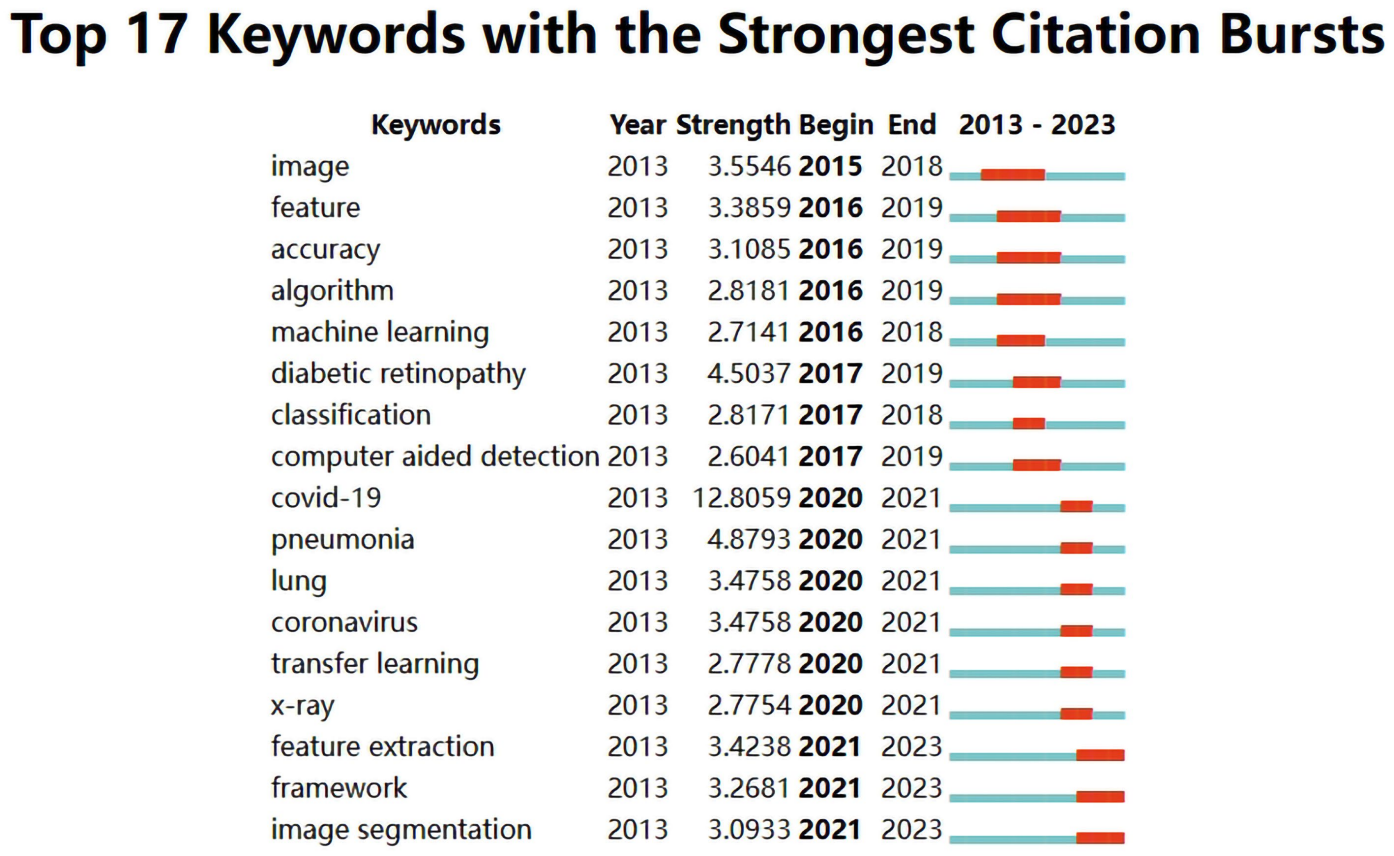
Top 17 keywords with the strongest citation bursts in publications of medical image processing with deep learning from 2013 to 2023. The blue line represents the overall timeline, while the red line represents the appearance year, duration, and end year of the burst keywords.

The evolution of burst keywords in recent years can be summarized as follow (Figure 6): It all began in 2015 with a focus on “image.” By 2016, “feature, accuracy, algorithm, and machine learning” took center stage. The year 2017 brought prominence to “diabetic retinopathy, classification and computer-aided detection.” Moving into 2020, attention shifted to “COVID-19, pneumonia, lung, coronavirus, transfer learning and X-ray.” In 2021, the conversation revolved around “feature extraction, framework and image segmentation”.

### 3.6. Analysis of references

**Figure 7:**
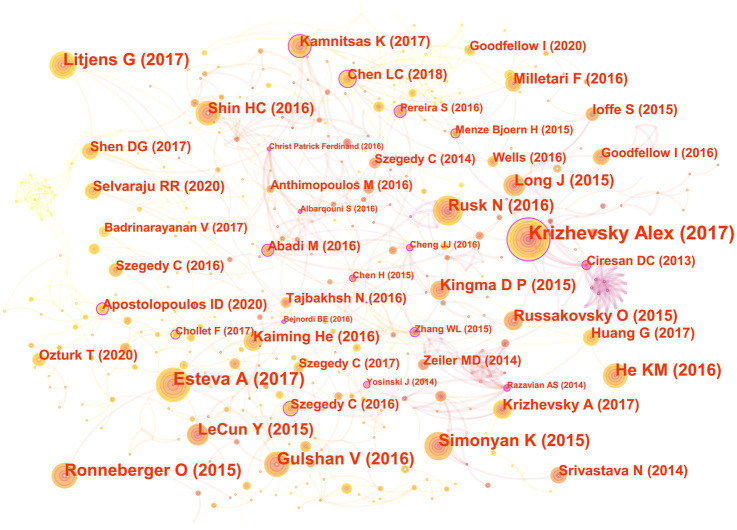
The co-cited reference map in the field of medical image processing with deep learning from 2013 to 2023. The size of nodes reflects the number of citations, while the links between nodes reflect the strength of co-citations.

In the 562 articles included, there are a total of 584 references (Figure 7; Table 4). The most cited reference is “ImageNet Classification with Deep Convolutional Neural Networks” by Alex Krizhevsky. Alex Krizhevsky and his team developed a powerful convolutional neural network (CNN) to classify a vast dataset of high-resolution images into 1,000 categories, achieving significantly improved accuracy rates of 37.5 and 17.0% for top-1 and top-5 errors compared to previous methods (Krizhevsky et al., 2017).

**Table 4:** Top 10 references in quantity ranking.

Rank	Number of citations	Title
1	117	ImageNet classification with deep convolutional neural networks
2	90	Dermatologist-level classification of skin cancer with deep neural networks
3	68	Very deep convolutional networks for large-scale image recognition
4	67	Deep residual learning for image recognition
5	67	U-Net: convolutional networks for biomedical image segmentation
6	66	Development and validation of a deep learning algorithm for detection of diabetic retinopathy in retinal fundus photographs
7	63	A survey on deep learning in medical image analysis
8	53	Deep learning
9	50	Deep learning
10	48	ImageNet large scale visual recognition challenge

There are a total of three articles with centrality greater than or equal to 0.1. The authors of these three articles are Dan Claudiu Ciresan, Liang-Chieh Chen, and Marios Anthimopoulos. Dan Claudiu Ciresan use deep max-pooling convolutional neural networks to detect mitosis in breast histology images and won the ICPR 2012 mitosis detection competition (Ciresan et al., 2013). Liang-Chieh Chen address the task of semantic image segmentation with deep learning and make three main contributions. Firstly, convolution with upsampled filters, known as “atrous convolution.” Secondly, they introduce the method of atrous spatial pyramid pooling (ASPP). Lastly, they improve the accuracy of object boundary localization by integrating techniques from deep convolutional neural networks and probabilistic graphical models (Chen et al., 2018). Marios Anthimopoulos propose and evaluate a convolutional neural network (CNN), designed for the classification of interstitial lung diseases (ILDs) patterns (Anthimopoulos et al., 2016).

The eighth and ninth ranked articles have the same title, originating from the Nature journal. The commonality lies in their source, but they differ in authors. The eighth-ranked article is by Nicole Rusk, published in the Comments & Opinion section of Nature Methods. It provides a concise introduction to deep learning (Rusk, 2016). On the other hand, the ninth-ranked article is authored by Yann LeCun and is a comprehensive review. In comparison to Nicole Rusk’s article, LeCun’s extensively elaborates on the fundamental principles of deep learning and its applications in various domains such as speech recognition, visual object recognition, object detection, as well as fields like drug discovery and genomics (LeCun et al., 2015).

### 3.7. Analysis of co-cited authors

**Figure 8:**
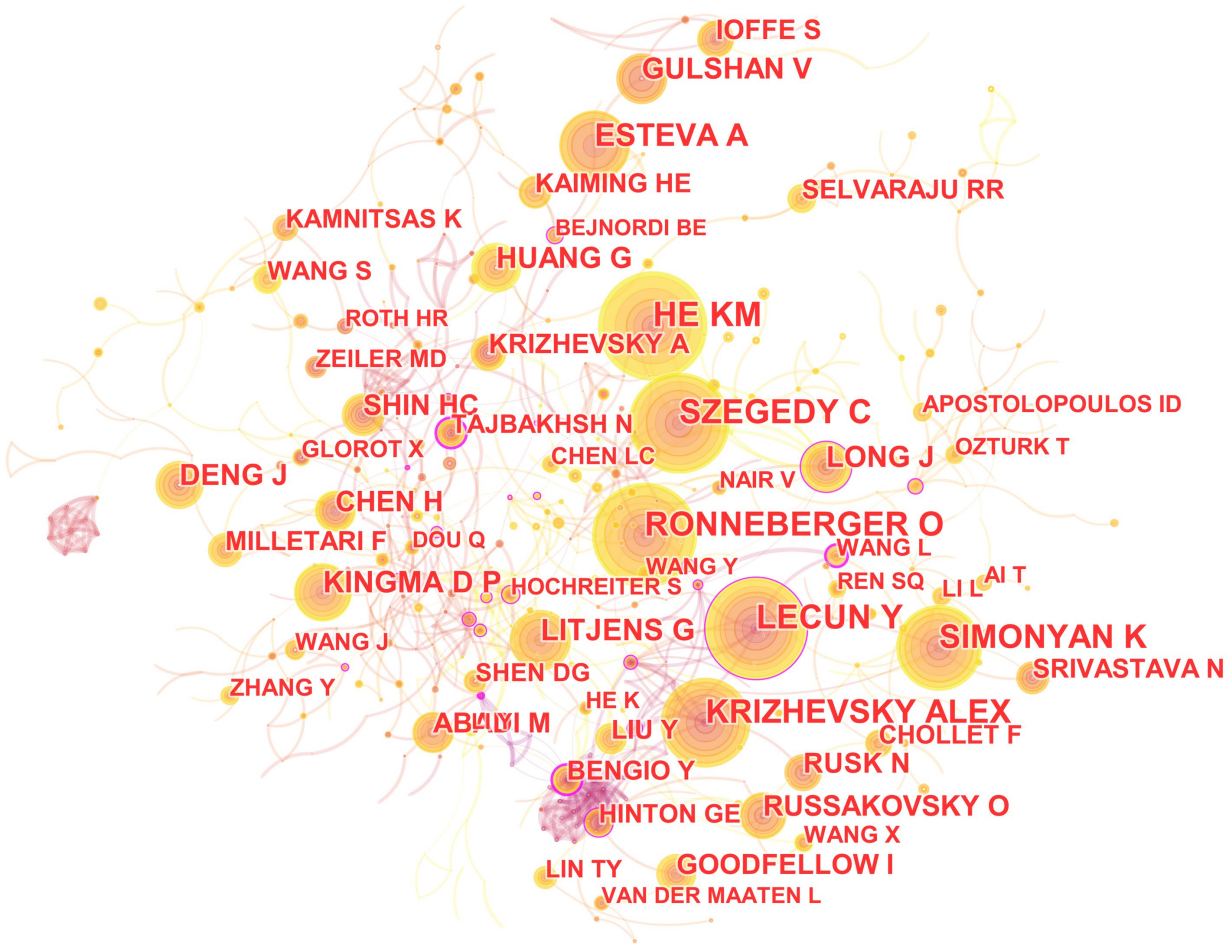
The map of co-cited author in the field of medical image processing with deep learning from 2013 to 2023. The size of nodes reflects the number of citations, while the links between nodes reflect the strength of co-citations.

In the 562 included articles, there are a total of 634 cited authors (Figure 8). The most cited author is Kaiming He, whose papers have been cited 141 times; the author with the highest centrality is Yoshua Bengio, whose papers have been cited 45 times.

The most cited paper authored by Kaiming He in Web of Science is “Deep Residual Learning for Image Recognition.” This paper introduces a residual learning framework to simplify the training of networks that are much deeper than those used previously. These residual networks are not only easier to optimize but also achieve higher accuracy with considerably increased depth (He et al., 2016). On the other hand, the most cited paper authored by Yoshua Bengio in Web of Science is “Representation Learning: A Review and New Perspectives.” This paper reviews recent advances in unsupervised feature learning and deep learning, covering progress in probabilistic models, autoencoders, manifold learning, and deep networks (Bengio et al., 2013).

### 3.8. Analysis of co-cited journals

**Figure 9:**
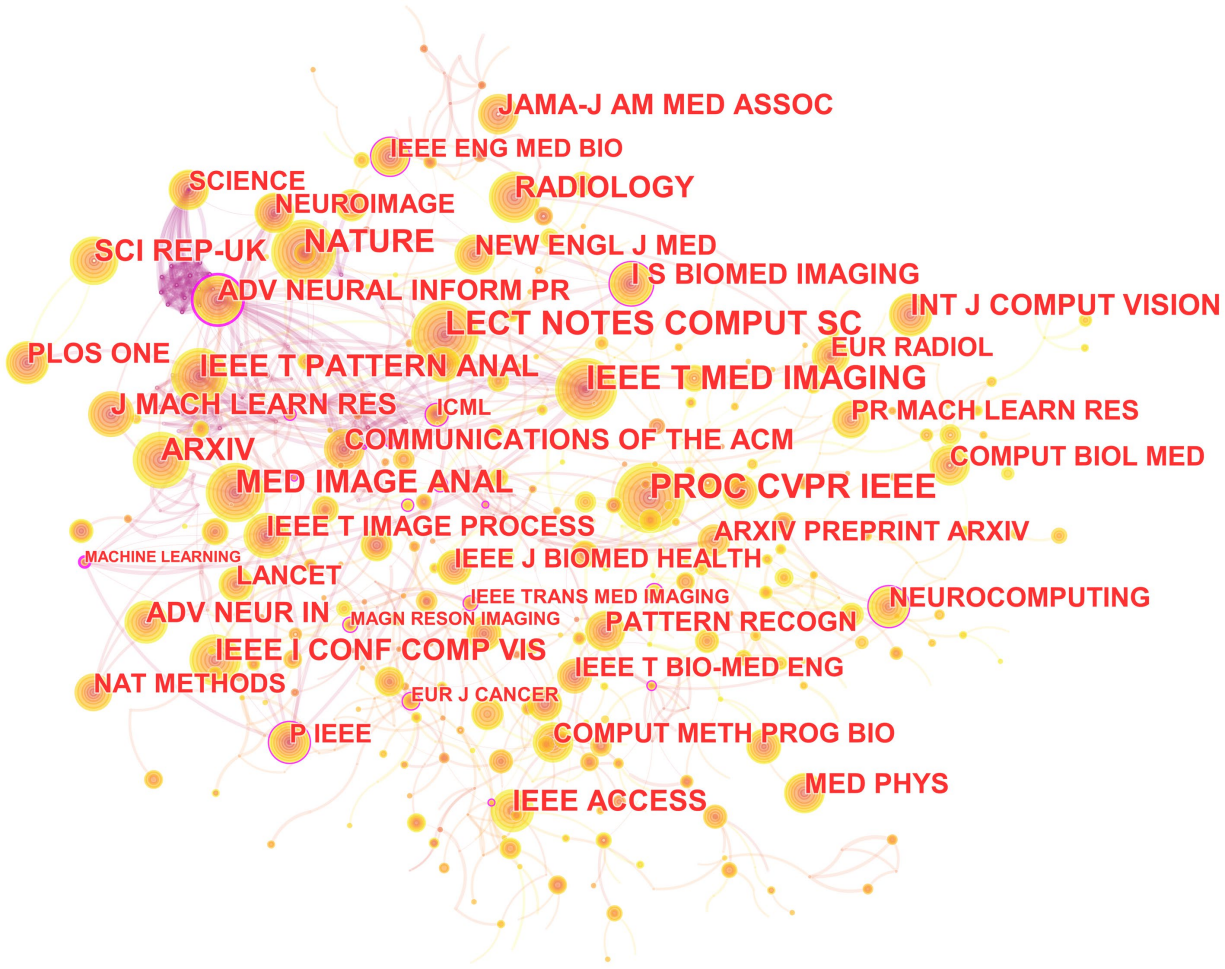
The collaborative relationship map of co-cited journal in the field of medical image processing with deep learning from 2013 to 2023.The size of nodes reflects the number of citations, while the links between nodes reflect the strength of co-citations.

In the 562 articles included, a total of 345 journals were cited (Figure 9; Table 5). The journal with the most citations is the IEEE Conference on Computer Vision and Pattern Recognition, with 339 articles citing papers from this journal; the journal with the highest centrality is Advances in Neural Information Processing Systems, with 128 articles citing papers from this journal.

**Table 5:** Top 10 journals in citation frequency and centrality ranking.

Rank	Number of citations	Journal	Centrality	Journal
1	339	IEEE Conference on Computer Vision and Pattern Recognition	0.3	Advances in Neural Information Processing Systems
2	312	Lecture Notes in Computer Science	0.22	Machine Learning
3	295	IEEE Transactions on Medical Imaging	0.18	IEEE Transactions on Neural Networks and Learning Systems
4	240	Medical Image Analysis	0.17	Alzheimer’s & Dementia
5	217	Nature	0.15	IEEE Winter Conference on Applications of Computer Vision
6	206	Arxiv	0.15	IEEE Transactions on Medical Imaging
7	179	IEEE Transactions on Pattern Analysis and Machine Intelligence	0.14	IEEE International Symposium on Biomedical Imaging
8	168	Radiology	0.14	Neurocomputing
9	163	IEEE International Conference on Computer Vision	0.14	European Journal of Cancer
10	149	Scientific Reports	0.13	IEEE Engineering in Medicine and Biology Magazine

**Figure 10:**
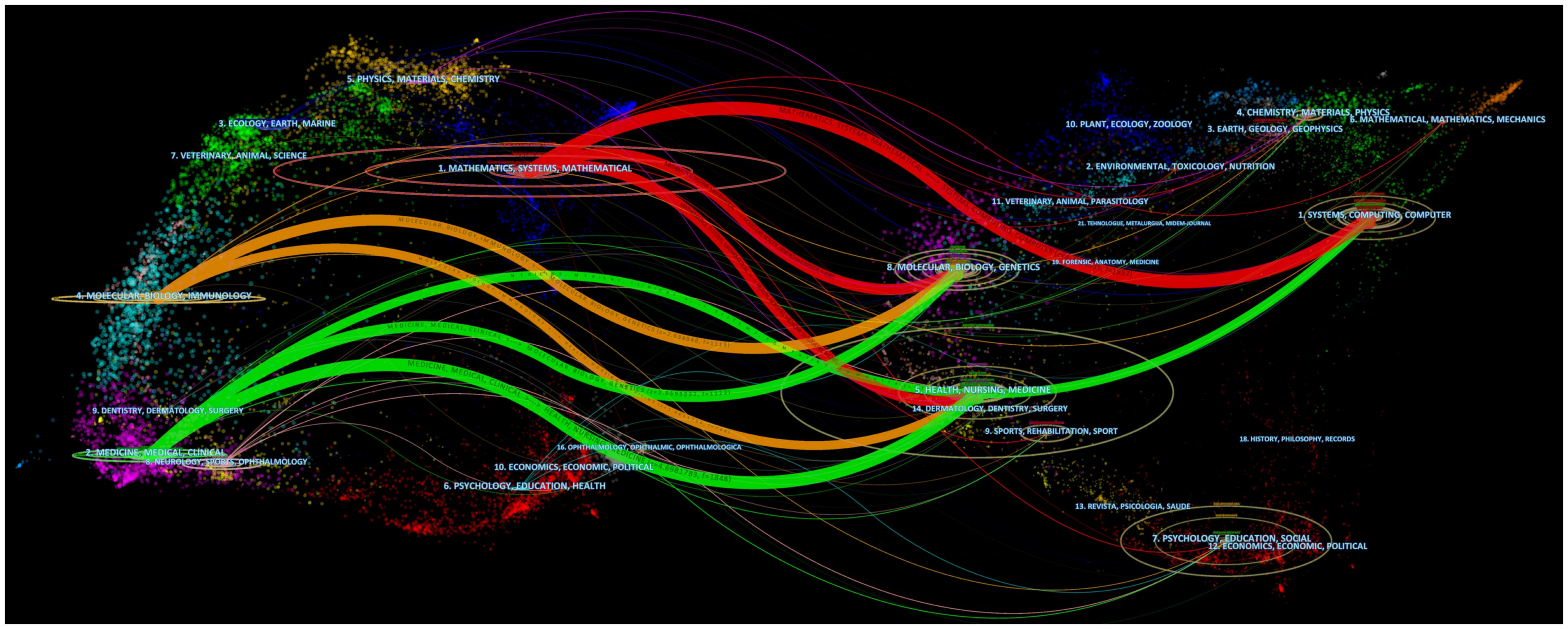
Dual-map overlap of journals. The map consists of two graphs, with the citing graph on the left and the cited graph on the right. The curves represent citation links, displaying the full citation chain. The longer the vertical axis of the ellipse, the more articles are published in the journal. The longer the horizontal axis of the ellipse, the more authors have contributed to the journal.

It can be seen that the literature in three major disciplines, mathematics, systems, and mathematical, cite systems, computing, computers; molecular, biology, genetics; health, nursing, and medicine. The literature in molecular, biology, and immunology cite molecular, biology, genetics, and literature in health, nursing, and medicine. The literature in medicine, medical, and clinical cite molecular, biology, genetics, and literature in health, nursing, medicine (Figure 10).

## 4. Discussion

From 2013 to 2023, the analysis of publication volume reveals an obvious stage characteristic, before and after 2016, and thus, 2016 is a key year for the field of deep learning-based medical image processing. Although deep learning technology began to be applied as early as 2012, it did not receive widespread attention in the field of medical image processing because traditional machine learning methods, such as support vector machines (SVM) and random forests (Lehmann et al., 2007), were mainly used before then. At the same time, deep learning models require powerful computing power and a large amount of data for training (Ren et al., 2022). Before 2016, high-performance computers were very expensive, which was not conducive to large-scale research in this field. Moreover, large-scale medical image datasets were relatively scarce, so research in this field was constrained by computing capability and dataset limitations. In 2016, however, deep learning technology achieved breakthroughs in computer vision, including image classification, object detection, and segmentation, providing more advanced and efficient solutions for medical image processing (Girshick et al., 2016; Madabhushi and Lee, 2016). These breakthroughs accelerated the progress of research in this field, leading to an increase in publication volume year by year.

From the analysis of authors, it can be seen that the research on deep learning in the field of medical image processing is relatively scattered, and large-scale cooperative teams have not been formed. This may be because research on deep learning requires a large amount of computing resources and data, and therefore requires a strong background in mathematics and computer science. At the same time, the application of deep learning in the medical field is an interdisciplinary cross, which also requires the participation of talents with medical backgrounds. However, individuals with both backgrounds are relatively few, making it difficult to form large-scale research teams. In addition, researchers in this field may be more focused on personal research achievements rather than collaborating with others. This situation may not necessarily mean that researchers lack a spirit of cooperation, but rather reflects the research characteristics and preferences of this field’s researchers.

The institutional analysis mainly reflects two characteristics: first, the broad cooperation between institutions is mainly based on high publication volume and high centrality institutions; publication volume and centrality are not necessarily correlated. This indicates that in the field of medical image processing, institutions with high publication volume and centrality often have strong collaborative abilities and influence, which can attract other institutions to cooperate with them. However, institutions with low publication volume and no centrality may collaborate less due to a lack of resources or opportunities. Second, publication volume does not entirely determine centrality. Sometimes smaller institutions may receive high attention and recognition due to their unique research contributions or research directions (Wuchty et al., 2007; Lariviere and Gingras, 2010). Therefore, institutional centrality is not only related to publication volume but also to the depth and breadth of research, and the degree of innovation in research results. Overall, these institutions are internationally renowned research institutions with broad disciplinary areas and research capabilities, and they have high centrality in the field of medical image processing, making them important research institutions in this field. The collaboration and communication between these institutions are also very frequent, jointly promoting the development of medical image processing. These institutions are distributed globally, including countries and regions such as China, the United States, Germany, and the United Kingdom, showing an international character. Among them, the United States has the largest number of institutions, occupying two of the top three positions, indicating that the United States has strong strength and influence in the field of medical image processing. In addition, these institutions include universities, hospitals, and research institutes, demonstrating the interdisciplinary nature of the field of medical image processing. These institutions also often collaborate and communicate with each other, jointly promoting the research progress in this field.

In country analysis, there are mainly three situations: some countries not only have a large number of publications, but also have high centrality; some countries have a small number of publications, but high centrality; and some countries have a large number of publications, but low centrality. This indicates that deep learning in medical image processing is a global research hotspot, and various countries have published high-quality papers in this field and have close collaborative relationships. Some countries have a large number of publications in this field because they have strong research capabilities and play a leading role in this field. The high centrality of these countries also indicates that they play an important role in collaborative relationships. Some countries have a relatively low number of publications, but their centrality is still high. This may be because they have unique contributions in specific research directions or technologies in this field (Lee et al., 2018), or because they have close relationships with other countries in this field. There are also some countries with a large number of publications, but low centrality. This may be because their research and published paper quality is relatively low in this field, or because they have relatively few collaborative relationships with other countries.

According to keyword analysis, these keywords indicate that in highly cited papers in the field of medical image processing, core concepts include deep learning and machine learning, such as “deep learning” and “machine learning.” In terms of applications, the keywords emphasize COVID-19 diagnosis, image segmentation, and classification, while highlighting the significance of neural networks and convolutional neural networks. Additionally, the centrality-ranked keywords underscore the relevance of algorithms associated with deep learning and reiterate key themes in medical image processing, such as “cancer” and “MRI.” Overall, these keywords reflect the diverse applications of deep learning in medical image processing and the importance of algorithms.

From the clusters of keywords, these clusters can be grouped into four main domains, reflecting diverse applications of deep learning in medical image processing. The first group focuses on medical image processing and diseases, encompassing biomarkers, the detection, and diagnosis of specific diseases such as breast cancer and COVID-19 (Chougrad et al., 2018; Altan and Karasu, 2020). The second group concentrates on image processing and computer vision, including image restoration, annotation, and change detection (Zhang et al., 2016; Kumar et al., 2017; Tatsugami et al., 2019) to enhance the quality and analysis of medical images. The third group emphasizes data analysis and information processing, encompassing feature learning, bioinformatics, and information extraction (Min et al., 2017; Chen et al., 2021; Hang et al., 2022), aiding in the extraction of valuable information from medical images. Lastly, the fourth group centers on neuroscience and medical imaging, studying brain networks and ultrasound images (Kawahara et al., 2017; Ragab et al., 2022), highlighting the importance of deep learning in understanding and analyzing biomedical images for studying the nervous system and organs.

From the analysis of burst keywords, the evolution of these keywords reflects the changing trends and focal points in the field of deep learning in medical image processing. In 2015, the keyword “image” dominated, signifying an initial emphasis on basic image processing and analysis to acquire fundamental image information. By 2016, terms like “feature,” “accuracy,” “algorithm,” and “machine learning” (Shin et al., 2016; Zhang et al., 2016; Jin et al., 2017; Lee et al., 2017; Zhang et al., 2018) were introduced, indicating a growing interest in feature extraction, algorithm optimization, accuracy, and machine learning methods, highlighting the shift toward higher-level analysis and precision in medical image processing. In 2017, terms like “diabetic retinopathy,” “classification,” and “computer-aided detection” (Zhang et al., 2016; Lee et al., 2017; Quellec et al., 2017; Setio et al., 2017) were added, underlining an increased interest in disease-specific diagnoses (e.g., diabetic retinopathy) and computer-assisted detection of medical images. The year 2020 saw the emergence of “COVID-19,” “pneumonia,” “lung,” “coronavirus,” “transfer learning,” and “x-ray” (Minaee et al., 2020) due to the urgent demand for analyzing lung diseases and infectious disease detection, prompted by the COVID-19 pandemic. Additionally, “transfer learning” reflected the trend of utilizing pre-existing deep learning models for medical image data. In 2021, keywords such as “feature extraction,” “framework,” and “image segmentation” (Dhiman et al., 2021; Sinha and Dolz, 2021; Chen et al., 2022) became prominent, indicating a deeper exploration of feature extraction, analysis frameworks, and image segmentation to enhance the accuracy and efficiency of medical image processing. Overall, these changes illustrate the ongoing development in the field of medical image processing, evolving from basic image processing toward more precise feature extraction, disease diagnosis, lesion segmentation, and addressing the needs arising from disease outbreaks. This underscores the widespread application and continual evolution of deep learning in the medical domain.

Based on the analysis of reference citations, it is evident that these 10 highly cited papers cover significant research in the field of deep learning applied to medical image processing. They share a common emphasis on the outstanding performance of deep Convolutional Neural Networks (CNNs) in tasks such as image classification, skin cancer classification, and medical image segmentation. They explore the effectiveness of applying deep residual learning in large-scale image recognition and medical image analysis (He et al., 2016). The introduction of the U-Net, a convolutional network architecture suitable for biomedical image segmentation, is another key aspect (Ronneberger et al., 2015). Additionally, they develop deep learning algorithms for detecting diabetic retinopathy in retinal fundus photographs (Gulshan et al., 2016). They also provide a review of deep learning in medical image analysis, summarizing the trends in related research (LeCun et al., 2015; Rusk, 2016). However, these papers also exhibit some differences. Some focus on specific tasks like skin cancer classification and diabetic retinopathy detection, some concentrate on proposing new network structures (such as ResNet, U-Net, etc.) to enhance the performance of medical image processing, while others provide overviews and summaries of the overall application of deep learning in medical image processing. Overall, these papers collectively drive the advancement of deep learning in the field of medical image processing, achieving significant research outcomes through the introduction of new network architectures, effective algorithms, and their application to specific medical image tasks.

From the analysis of cited journal, it can be observed that these journals collectively highlight the important features of research in medical image processing. Firstly, they emphasize areas such as computer vision, image processing, and pattern recognition, which are closely related to medical image processing. Moreover, journals and conferences led by IEEE, such as IEEE Transactions on Neural Networks and Learning Systems, IEEE Transactions on Medical Imaging, and IEEE Winter Conference on Applications of Computer Vision, hold significant influence in the fields of computer vision and pattern recognition, reflecting IEEE’s leadership in the domain of medical image processing. These journals span across multiple fields including computer science, medicine, and natural sciences, underscoring the interdisciplinary nature of medical image processing research. Open-access publishing platforms like Arxiv and Scientific Reports underscore the importance of open access and information sharing in the field of medical image processing. Additionally, specialized journals like “Medical Image Analysis” and “Radiology” play pivotal roles in research on medical image processing. The comprehensive journal “Nature” covers a wide range of scientific disciplines, potentially including research related to medical image processing. In summary, these journals collectively form a comprehensive research network covering various academic disciplines in the field of medical image processing, emphasizing the significance of open access and information sharing. They also highlight the crucial role of deep learning and neural network technologies in medical image processing, as well as the importance of image processing, analysis, and diagnosis.

From the analysis of dual-map overlap of journals, it can be observed that a particularly noteworthy citation relationship is the reference of computer science, biology, and medicine to mathematics. Computer science research has a strong connection to mathematics, as mathematical methods and algorithms are the foundation of computer science, while the development of computers and information technology provides a broader range of applications for mathematical research (Domingos, 2012). Molecular biology and genetics are important branches of biological research, where mathematical methods are widely applied, such as for analyzing gene sequences and molecular structures, and studying interactions between molecules (Jerber et al., 2021). Medicine is a field related to human health, where mathematical methods also have many applications, such as for statistical analysis of clinical trial results, predicting disease risk, and optimizing the allocation of medical resources (Gong and Tang, 2020; Wang et al., 2021).

From our perspective, the future development of deep learning in the field of medical image processing can be summarized as follows. First, with the widespread application of deep learning models in medical image processing, the design and development of more efficient and lightweight network architectures will become necessary. This can improve the speed and portability of the model, making it possible for these models to run effectively in resource-limited environments such as mobile devices (Ghimire et al., 2022). Second, traditional deep learning methods usually require a large amount of labeled data for training, while in the field of medical image processing, labeled data is often difficult to obtain. Therefore, weakly supervised learning will become an important research direction to improve the model’s performance using a small amount of labeled data and a large amount of unlabeled data. This includes the application of techniques such as semi-supervised learning, transfer learning, and generative adversarial networks (Ren et al., 2023). Third, medical image processing involves different types of data such as CT scans, MRI, X-rays, and biomarkers. Therefore, multimodal fusion will become an important research direction to organically combine information from different modalities and provide more comprehensive and accurate medical image analysis results. Deep learning methods can be used to learn the correlations between multimodal data and perform feature extraction and fusion across modalities (Saleh et al., 2023). Finally, deep learning models are typically black boxes, and their decision-making process is difficult to explain and understand. In medical image processing, the interpretability and reliability of the decision-making process are crucial. Therefore, researchers will focus on developing interpretable deep learning methods to enhance physicians’ and clinical experts’ trust in the model’s results and provide explanations for the decision-making process (Chaddad et al., 2023).

In conclusion, deep learning is becoming increasingly important in the field of medical image processing, with many active authors, institutions, and countries in this field. In the high-cited papers of this field in the core collection of Web of Science, Pheng-Ann Heng, Hao Chen, and Dinggang Shen have published a relatively large number of papers. China has the most research institutions in this field, including the Chinese Academy of Sciences, the University of Chinese Academy of Sciences, The Chinese University of Hong Kong, Zhejiang University, and Shanghai Jiao Tong University. The United States ranks second in terms of the number of institutions, including Stanford University, Harvard Medical School, and Massachusetts General Hospital. Germany and the United Kingdom have relatively few institutions in this field. The number of publications in the United States far exceeds that of other countries, with China in second place. The number of papers from the United Kingdom, Germany, Canada, Australia, and India is relatively high, while the number of papers from the Netherlands and France is relatively low. South Korea’s development and publication output in medical image processing are relatively low. Currently, research in this field is mainly focused on deep learning, convolutional neural networks, classification, diagnosis, segmentation, algorithms, artificial intelligence, and other aspects, and the research focus and trends are gradually moving toward more complex and systematic directions. Deep learning technology will continue to play an important role in this field.

This study has certain limitations. Firstly, we only selected highly cited papers from the Web of Science Core Collection as our analysis material, which means that we may have missed some highly cited papers from other databases and our analysis may not be comprehensive for the entire Web of Science. However, given the limitations of bibliometric software, it is difficult to merge and analyze various databases. Additionally, the reasons why we chose highly cited papers from the Web of Science Core Collection as our analysis material have been explained in the section “Introduction.” Secondly, we may have overlooked some important non-English papers, leading to research bias.

## Data availability statement

The original contributions presented in the study are included in the article/supplementary material, further inquiries can be directed to the corresponding authors.

## Author contributions

BC: Writing – original draft. JJ: Writing – review & editing. HL: Writing – review & editing. ZY: Writing – review & editing. HZ: Writing – review & editing. YW: Writing – review & editing. JL: Writing – original draft. SW: Writing – original draft. SC: Writing – original draft.
